# Involuntary psychiatric treatment and the erosion of consent: A critical discourse analysis of mental health legislation in British Columbia, Canada

**DOI:** 10.1177/13634593221096241

**Published:** 2022-05-09

**Authors:** Maja Kolar, Colleen Varcoe, Helen Brown, Rochelle Einboden

**Affiliations:** School of Nursing, University of British Columbia, Canada; Susan Wakil School of Nursing and Midwifery, The University of Sydney, Faculty of Medicine and Health, Australia

**Keywords:** critical discourse analysis, mental health, legislation, consent, involuntary psychiatric treatment

## Abstract

The Mental Health Act (1996) is legislation that directs voluntary and involuntary psychiatric treatment for people experiencing mental health issues in British Columbia (BC), Canada. This critical discursive analysis explores how BC’s Mental Health Act (1996) and the Guide to the Mental Health Act (2005) structure involuntary psychiatric treatment and illustrates how the discourses within these texts constitute people experiencing mental health issues as passive recipients of care. Understandings of people experiencing mental health issues as pathological, incapable, vulnerable and dangerous justify their need for protection and the protection of others. Protection is identified as a central legitimising discourse in the use of involuntary psychiatric treatment. Further, these texts define the roles and responsibilities of police, physicians and nurses in authorising and implementing involuntary psychiatric treatment. This analysis describes how this legislation erodes consent and entrenches social marginalisation. Alternatively, discourses of equity have potential to transform health care practices and structures that reproduce discourses of deficit, vulnerability and dangerousness, shifting towards promotion of the rights and safety of people experiencing mental health issues and crises.

## Introduction

The Mental Health Act (1996) is a key influence on the nature and frequency of involuntary treatment for people experiencing mental health issues in British Columbia (BC), Canada. The primary purpose of this act is to ensure the supervision, protection and care of people experiencing mental health issues through the provision of ‘authority, criteria and procedures for involuntary admission and treatment’ (Ministry of Health, 2005: 1). The Guide to the Mental Health Act (2005) has been developed by the BC Ministry of Health to support patients, families, health care providers and police to understand and apply the act. Together these texts position involuntary psychiatric treatment as essential to mitigate substantial risk of deterioration and harm to self or others specifically for people experiencing impaired insight or an unwillingness to accept treatment voluntarily (Ministry of Health, 2005: 72).

Involuntary psychiatric treatment purports to reduce harm, yet BC legislation has been criticised by people with lived experience, their families, clinicians and advocates for issues concerning consent, decision-making authority, and coercive treatment practices (Health Justice, 2021; Johnston, 2017; Nurses and Nurse Practitioners of BC, 2021). In the context of involuntary treatment, consent to any psychiatric treatment deemed appropriate by the treating physician is assumed. The use of this model of ‘deemed consent’ combined with a lack of oversight of involuntary treatment has meant that BC is considered ‘the most regressive jurisdiction in Canada for mental health detention and involuntary psychiatric treatment’ (Johnston, 2017: 6). BC continues to face concerns with a lack of accessible and affordable voluntary treatment options for people experiencing mental health issues (Canadian Mental Health Association, 2018; Health Justice, 2021). Additionally, there are three key social issues impacting the situation for people experiencing mental health issues in BC: first, a housing crisis and rising levels of homelessness (Canadian Mental Health Association, 2018); second, an opioid crisis, with an epidemic of fatalities due to respiratory failure from substances tainted with fentanyl (Tyndall, 2018); third, the COVID-19 pandemic that has exacerbated mental health issues and substance use (Mental Health Commission of Canada, 2020). Failing to increase supports in response to these social concerns, the province has seen rising rates of criminalisation of persons experiencing mental health issues and police intervention using excessive force (Parent, 2011).

At the same time, involuntary treatment has become the primary means of providing psychiatric care in BC. In a landmark report, Johnston (2017) identified admission for involuntary psychiatric treatment in BC’s mental health institutions has risen approximately 70% over the last 10 years, whereas voluntary admissions have decreased in relation to population growth (Johnston, 2017; Office of the Ombudsperson, 2019). This disproportionate increase in involuntary admissions suggests an ever-increasing adversarial approach to treatment of people experiencing mental health issues (Health Justice, 2021; Johnston, 2017). Involuntary treatment in the community context, described in the Mental Health Act (1996) as ‘extended leave’, has nearly tripled since the Ministry of Health began tracking these rates approximately 15 years ago (Johnston, 2017). Extensive individual, social and economic costs of involuntary treatment implore that these trends be better understood. A critical examination of BC’s *Mental Health Act* offers the opportunity to analyse how legislation shapes involuntary psychiatric treatment.

## Methodology

In the context of dominance of involuntary treatment in BC’s mental health care system, the orientation of critical discourse analysis (CDA) to social justice offers a fitting analytic approach. CDA’s strength to identify and challenge ideologies embedded within discourses that extend inequity guides our departure from a focus on the individual to a broader view of how the act operates within the social context. In contrast to the use of the terms ‘mental disorder’ or ‘mental illness’ in the texts (Mental Health Act, 1996: 2), we use ‘mental health issues’, to acknowledge social and structural influences on a person’s mental health and well-being.

Fairclough’s (2009) dialectical relational approach to CDA offers a structure for analysis by investigating discourses within legislation that shape mental health care systems. CDA understands discourse as ‘semiotic ways of constructing aspects of the world (physical, social or mental) which can generally be identified with different positions or perspectives of different groups of social actors’ (Fairclough, 2009: 232). Discourse is shaped by the power, values and belief systems of a particular socio-historical context in which it is produced, distributed and consumed (Fairclough, 2009). Discourses construct understandings of and responses to mental health issues. Analysis focuses on the social context and the operations of the discourses in social practice. The central research questions of this analysis ask: how are people experiencing mental health issues discursively constituted? and, how do these discourses organise responses of health care providers? Analysis was conducted during research training by the first author who is an advanced practice psychiatric nurse in BC, and was supported by a supervision team with expertise in discourse analysis and equity-oriented research (other authors).

### Theoretical framework

Critical social theory provides further support for analysis of power (Van Dijk, 2001). A critical social theory informed approach to discourse analysis sees communication (spoken, written and/or visual) as actively constructing, reproducing, reflecting and transforming society through ideology and power relations (Evans-Agnew et al., 2016; Fairclough et al., 2011). These perspectives offer an approach to analyse and expose ‘structural relationships of dominance, discrimination, power and control as manifested in language’ (Moran and Wright, 2001: 2), which shape mental health care legislation and systems.

### Data corpus

The data corpus includes specific sections and forms from BC’s Mental Health Act (1996) and the Guide to the Mental Health Act (2005), which were selected for analysis due to their significant authority and influence over the involuntary treatment process. The act provides definitions for terms and guidance on its implementation, and is supported by 21 different forms. To admit a person for involuntary psychiatric treatment, it is a legal requirement to complete five of these forms, including: a medical certificate that outlines four criteria that the person must meet to be certified; a treatment plan; a notification of involuntary patient rights; a nomination of a relative; and a notification to the nominated relative of the person’s involuntary admission to hospital (Mental Health Act, 1996; Office of the Ombudsperson [British Columbia, 2019). In addition, four forms and four sections were included in the data corpus that provide instructions for: placing a patient on extended leave; apprehending a patient via warrant; police intervention; and liability. Although the Guide to the Mental Health Act (2005) is optional for individual providers to use in practice, it was included in the corpus because it is meant to reduce the amount of inferential work required to enact the legislation (Ministry of Health, 2005). The guide was first published in 1997, revised in 1999 and 2005, with a current revision underway. Detailed descriptions of the data corpus texts can be found in Table 1. All documents are publicly accessible online on BC’s government website.

**Table 1.** The data corpus.

Original Source	Content	Type of Text	Purpose of Text
*Mental Health Act* [RSBC, 1996] CHAPTER 288	• Part 1—Interpretation • Part 2—Administration • Part 3—Admission and Detention of Patients • Part 4—Regulations	Legislation	The Mental Health Act (1996) is legislation that provides interpretations of definitions for terms used within the act and guidance on implementation of the act.
Mental Health Act (1996)	• Form 4 Medical Certificate (Involuntary Admission) • Form 5 Consent for Treatment (Involuntary Patient) • Form 6 Medical Report on Examination of Involuntary Patient (Renewal Certificate) • Form 13 Notification to Involuntary Patient of Rights under the *Mental Health Act* • Form 15 Nomination of Near Relative • Form 16 Notification to Near Relative (Admission of Involuntary Patient or Patient Under Age 16) • Form 17 Notification to Near Relative (Discharge of Involuntary Patient) • Form 19 Certificate of Discharge • Form 20 Leave Authorisation • Form 21 Director’s Warrant (Apprehension of Patient) • Section 3 BCACP Police Triage Guide • Section 16 Protection from Liability • Section 22 Involuntary Admissions • Section 28 Police Intervention	Mental Health Act (1996) forms	The Mental Health Act (1996) forms are the manner in which the act is applied by health care providers and police officers.
Guide to the Mental Health Act (2005)	• Sections pertaining to the selected Mental Health Act (1996) forms within the Guide to the Mental Health Act (2005): pp. 1–94	Supporting document to the Mental Health Act (1996)	The Guide to the Mental Health Act is a tool developed by the Ministry of Health (2005) to help translate the act for patients, families and health care providers.

### Data analysis

The act and guide are characterised by usual features of texts within the legal genre, in that they follow authoritative patterns, are conservative, slow to change, precedent setting, formal and use detailed language (Fairclough, 1992). However, CDA makes it possible to see how legislation can go beyond operating prescriptively. Legal texts can be understood for how they both sustain and reflect social values and beliefs, and how resulting discourses legitimise relations of power (Fiske and Browne, 2008 ). Thus, dialectical relations are seen between discourse and relations of power, as well as social values and beliefs.

Analysis for this study follows Fairclough’s (2009) four stages of analysis outlined in Table 2. While these stages offer a framework, they operate in an iterative rather than linear manner. In the first stage, the ‘social wrong’ was identified *as the erosion of consent for people experiencing mental health issues*. Analysis at this stage focuses on identification of discourses and a socio-historical overview of the legislation in BC, in the context of deinstitutionalisation. In the second stage, an overview of the texts within the data corpus was conducted, including identifying discourses, interdiscursivity, naturalisations, social relations, representations and identities, as well as interpretive implications (Fairclough, 1992). How these discourses form obstacles to addressing erosion of consent is considered. In the third stage, how involuntary psychiatric treatment supports the current social order is considered. In the fourth stage, alternatives to dominant conceptualisations of, and approaches to, mental health care are explored. Orientation of this analysis towards social justice also points to possibilities for legislative reform, and holds promise to rebuild the importance of consent for people experiencing mental health issues or crises in BC.

**Table 2.** Stages of dialectical-relational critical discourse analysis.

Analytical Stage	Description
Stage 1: Describe the social wrong and its semiotic aspects	This analysis focussed on the social wrong being the erosion of consent towards people experiencing mental health issues identified as requiring of involuntary treatment. Specifically, we examined how the act and guide shape involuntary treatment and positions involuntary patients, including those who enact the act (such as physicians, nurses and police officers).
Stage 2: Identify obstacles to addressing the social wrong	Within this stage the obstacles to addressing dominant discourses within the object of analysis are investigated. We considered the social and historical influences that shaped the conceptualisation of ‘mental disorders’, including the role of biomedicine, medical authority, psychiatry, ableism and the positioning of people experiencing mental health issues. Within this stage the selected texts were analysed and findings from this stage identified two discourses, ‘the involuntary patient’ and ‘the need for protection’.
Stage 3: Consider whether the social order ‘needs’ the social wrong	This stage explored how the current social order ‘needs’ the social wrong. The social wrong involves the problematic positioning of the patient and the outcomes of this positioning, being the exacerbation of social and economic marginalisation. The authors analysed how involuntary treatment functions to centralise control and maintain the dominance of biomedicine, as well as the individualising of people experiencing mental health issues as pathological, obscuring the need to address structural determinants of mental health such as housing and income.
Stage 4: Identification of possible ways past the obstacles	In this stage the analysis focussed on identifying alternatives to dominant conceptualisations and approaches to mental health practice and treatment, and how these alternatives might reduce coercion.

Instead of traditional distinctions between results and discussion, this paper is organised discursively. First, the history and contexts in which the act was produced is discussed, followed by discussion of two central discourses: ‘the involuntary patient’ and ‘the need for protection’. Next, how these discourses shape the roles of service providers is explored, followed by consideration of the operations of the act in health care settings. Ideologies and power relations sustained by this legislation, along with their impact on mental health practice are discussed. Finally, application of this analysis to suggested possibilities for legislative reform are presented.

## BC’s *Mental Health Act*: the history and context of deinstitutionalisation

In the late 1960′s to 1970′s, disability rights activist groups argued for transitioning people experiencing mental health issues from institutions to community-based care in response to identified gross human right violations, including sterilisation, segregation (promoted via eugenics), confinement, abuse, experimentation, overcrowding and unsanitary conditions within institutions (Yearwood-Lee, 2008). BC’s first *Mental Health Act* was established in 1964 within this context of closure of these institutions, the development of psychotropic medications, and the government’s desire to reduce expenditures (Dyck, 2011). The *Mental Health Act* was the product of a consolidation of five legislative acts: *Clinics of Psychological Medicine; Schools for Mental Defectives Act; Mental Hospitals Act; Provincial Mental Health* Centres *Act;* and *Provincial Child Guidance Clinics Act* (Government of British Columbia, 1964 ). Prior to this consolidation, people experiencing mental health issues in BC were admitted for involuntary treatment through physician’s orders and judicial endorsement (Fraser, 2015).

Dominant medical discourses of psychopathology underpin this legislation. Drawing on the *Diagnostic and Statistical Manual of Mental Disorders* (Ministry of Health, 2005: 1), a ‘mental disorder’ is understood as ‘a disorder of the mind that requires treatment and seriously impairs [a]. . . person’s ability to react appropriately to [their]. . . environment, or to associate with others’ (Ministry of Health, 2005: 72). Standardised diagnoses using a common classification system facilitate shared understandings among health care providers and promote a uniform approach to treatment (Lempérière, 1995). Diagnosis also assists in obtaining state funded disability assistance, incentivising practitioners to diagnose and people experiencing mental health issues to seek diagnosis (Brinkmann, 2016). While biomedical perspectives constitute ‘abnormal’ psychological experiences resulting from neurological deficits or genetic abnormalities that necessitate pharmaceutical intervention (Abramowitz, 2015 ; Deacon, 2013), diagnoses are underpinned by behaviour evaluated against socially accepted norms. Individuals who *behave* in ways that are outside of what is defined as ‘normal’ are constituted as pathological (Rose, 2006 ).

Similar to legislation in other Canadian provinces at the time, BC’s legislation was identified as utilising the “‘beneficence’ model of mental health care”, relying upon medical experts for decision-making (Fraser, 2015: 52). These paternalisms were entrenched through significant amendments in 1998, which allowed ‘psychiatrists to impose involuntary . . . treatment on patients whom they believe[d . . . were] at risk of deteriorating without medication, even though the patients [may be . . .] mentally capable of making treatment decisions for themselves and [. . .] not at risk of harming themselves or others’ (Fraser, 2015: 59). These changes to the legislation occurred at a time of ‘remarkable growth in psychiatric power and the sharp increase in the use of psychiatric medication’ solidifying ‘neurobiological forms of thinking shap[ing] the law, which then in turn codified that thinking and reinforced it through regulations, directives and documentary forms used by psychiatrists, hospitals and law enforcement agencies’ (Fraser, 2015: 59–60). Only minor amendments to the act have occurred since 1998, with structural revisions to forms currently underway (Government of British Columbia, 2019; Health Justice, 2021).

Dyck (2011) argues that deinstitutionalisation is a misleading description of what has happened with mental health services in BC and suggests a more accurate description is of ‘transinstitutionalisation’. Despite goals of rehabilitation and reintegration of people experiencing mental health issues into the community setting, there has been a high frequency of relapse, reliance on emergency services, readmission to hospital, and increased homelessness, crime and incarceration (Senate Canada, 2004). While inadequate resource allocation and lack of continuity of care are often cited as the primary issues contributing to poor outcomes, McWade (2016) cautions that these arguments ignore underlying structural issues and practices of psychiatry.

The community model remains the primary approach to mental health treatment across Canada, however specialised hospital psychiatric units offer brief intervention, treatment and stabilisation, with longer term treatment in tertiary centres or programmes. Thus, institutional care continues with a shift in the onus for ensuring access to care ‘from the state and medical authorities to consumers, patients and families who needed to navigate the contours of a patchwork of services, supports, and gaps in a modern health system’ (Dyck, 2011: para 7). In this milieu, psychiatric treatment continues to negotiate ‘the line between social support and coercive control’ (Boyd and Kerr, 2016: 425; Morrow et al., 2008), with the extension of paternalistic care into BC’s community settings under extended leave directives.

## Constitutions of the ‘Involuntary Patient’: Vulnerable and dangerous

Involuntary psychiatric treatment under BC’s *Mental Health Act* describes the process whereby ‘a person with a mental disorder or as apparently a person with a mental disorder’ is ‘detained or taken charge of’ for purposes of ‘receiving care, supervision, treatment, maintenance or rehabilitation’ (Mental Health Act, 1996: 2), and ‘control’ (Mental Health Act, 1996: s. 22). A person must be assessed and certified by a physician to be detained in a designated facility, or recalled from extended leave, to prevent ‘substantial mental or physical deterioration’ or to protect from self-harm and/or for the protection of others (Mental Health Act, 1996: s. 2; Ministry of Health, 2005). Once admitted to a designated facility, the person is identified as an ‘involuntary patient’ (Ministry of Health, 2005: 2). As an ‘involuntary patient’ the act allows treatments as ordered by the physician and authorised by the director – regardless of the patient’s capacity to consent to specific treatments and/or comprehend their benefits and risks (Ministry of Health, 2005: 2).

In these texts, persons in need of involuntary treatment are discursively constructed as disordered and vulnerable. In being constructed as incapable of caring for themselves and lacking agency to engage in decision-making about treatment, limited attention is paid to their preferences, resources or agency. Persons who require involuntary treatment are furthermore constituted by way of the definition in *Section 28 (1): Apprehension by Police*, as a dangerous person who is ‘apparently suffering from [a] mental disorder’ and ‘is acting in a manner likely to endanger their own safety or that of others’ (Mental Health Act, 1996: s.28). As per the BC *Association of Chiefs Police Triage Guide* referenced in the guide, this includes behaviours such as ‘unprovoked threats of violence to self or others; causing or inviting unprovoked serious injury or damage to self or others, habitual uncontrolled risks to physical safety or well-being of self/others; desire to seek revenge against “enemies”’ (Ministry of Health, 2005: 187). Lastly, identification as ‘patient’ connotes a unilateral relationship between the person and their health care provider, clarifying roles and responsibilities. The patient is the passive recipient, who patiently relies on diagnosis and treatment decisions made by the experts (Nair et al., 2000).

Since biomedicine defines mental health as the absence of illness or disease, emphasis remains centred on individual cure ‘as the measure of success’ (Pauly, 2008; Storch et al., 2002: 210). Diagnostic practices have been critiqued for locating problems within individuals (Kinderman and Cooke et al., 2017). A person experiencing ‘a disorder of the mind’ (Ministry of Health, 2005: 72) is deemed less capable of contributing to society, thus less valuable or even as social liability in comparison to an unaffected or non-psychitrised person. The focus on individuals rather than structures exacerbates social marginalisation experienced by people who are more likely to face disproportionate levels of involuntary psychiatric treatment (Czyzewski, 2011; Drescher, 2010; Kanani, 2011; Livingston, 2013; Walker, 2006).

This biomedical model fails to address underlying social issues and inequities that are ‘avoidable, socially produced and structurally driven’ (Boyd and Kerr, 2016; Ingram et al., 2013: 8). Concentrating on the individual diverts attention away from the social contexts, despite that these contexts have a profound influence on mental health (e.g. poverty, homelessness, ableism, colonialism, criminalisation, racism, transphobia, homophobia, lack of accessible voluntary and preventative services) (Bryant et al., 2019; Health Justice, 2021). Without appreciating social influences, dominant approaches to treatment continue to reinforce systemic barriers and undermine prevention-oriented services that prioritise collaboration with people seeking intervention and care (Shier et al., 2011). With limited preventative care options and inequitable access to social determinants of health, a person’s mental state may deteriorate to a point where the violation of their freedoms is required to receive psychiatric treatment within BC’s mental health system (Canadian Mental Health Association, 2017).

People who are more likely to be certified under the act include those who are less able to access voluntary treatment options. Inequities in access to voluntary care may be due to poverty, living in remote areas, substance use, or racism, ableism, ageism, classism, or other forms of discrimination such as sexual orientation or gender identity (Czyzewski, 2011; Drescher, 2010; Kanani, 2011; Livingston, 2013; Walker, 2006). Discrimination has legitimised intrusive and non-consensual medical interventions throughout history, and continues to act as justifications for involuntary psychiatric treatment practices (Czyzewski, 2011; Drescher, 2010; Kanani, 2011; Livingston, 2013; Walker, 2006). While perceptions of people experiencing mental health issues have changed overtime, socio-historic contexts of discrimination continue to intersect with involuntary treatment practices, deepening systemic discrimination against persons experiencing mental health issues.

## The need for ‘protection’

The discourse of protection figures centrally through the texts in relation to patients, others (the public), and liability. While the texts do not provide a definition of protection, the concept and practice of protection is constructed in a specific manner (Ministry of Health, 2005). The guide references the BC Supreme Court case of McCorkell v. Riverview Hospital (1993) for the interpretation of need for protection that goes beyond that of physical dangerousness and includes: ‘threats, violence, paranoid delusions, command hallucinations, irrational wasting of money, deteriorating physical condition, likelihood of or losing a job, dropping out of school, grossly unsanitary living conditions, and suicidal ideas or behaviours’ (Ministry of Health, 2005: 67). Such a broad interpretation of protection legitimises involuntary psychiatric treatment, and centralises decision-making with health care providers.

Aligning ‘mental disorders’ with incompetence and incapacity, protection of the patient includes monitoring and managing patient behaviour, even if it requires overriding consent or enforcing treatments (Ministry of Health, 2005: 1). With no references made in the act to engaging the patient’s perspectives or preferences, and only briefly mentioned in the guide, treatment decisions do not require direct consultation with people receiving care (Ministry of Health, 2005). The act and the guide leave the determination of need for protection open to interpretation and safeguards those implementing involuntary psychiatric treatment from legal liability for any harms occasioned during care (Mental Health Act, 1996: 6). The act defines the range of compulsory treatments for involuntary patients as inclusive of ‘any procedure necessarily related to the provision of psychiatric treatment’ (Mental Health Act, 1996: 3). Physicians are positioned with decision-making authority; they decide the course and type of treatment, and order measures to enforce compliance, including chemical/physical restraints or the use of seclusion. They determine the duration of time that protection is needed and set conditions for behaviour that must be met during treatment while admitted in an inpatient setting or on extended leave in the community. The dominance of psychiatric (biomedical) understandings of mental health treatment encourages a focus on diagnosis, pharmaceutical and medical interventions (Goulter et al., 2015).

Despite treatment being enacted in the ‘best interest’ of the patient (Ministry of Health, 2005: 43), there is silence on best interest in the act, and is only briefly mentioned a few times in the guide. Given the assumed vulnerability that underlies the reasoning for involuntary psychiatric treatment, the absence of definition and discussion of best interests is an important finding. Acting in a person’s best interest ‘paternalistically displac[es]. . . the actual or presumed views of the patient’ (Gurnham, 2008: 274). In contrast, the statement ‘best interest’ appears repeatedly in BC’s *Child, Family and Community Services Act (CFCSA)*, with paternalism obscured by a common-sense notion that adults should determine a child’s best interests on their behalf (Einboden et al., 2013). The *absence* of the mention of best interests in the *Mental Health Act* obscures paternalism, placing it out of view and beyond criticism. The lack of consent, accountability and patient engagement affords health care providers near unilateral power over involuntary patients. Involuntary psychiatric treatment creates a power relation whereby the patient ‘regulates their own conduct in accordance with the norms promoted by psychiatry and mental health nursing’ (Roberts, 2005: 36).

## Roles of police, physicians, and nurses in protection

*Section 28: Police Intervention* describes the role of police to apprehend people in public spaces who are ‘apparently suffering from [a] mental disorder’ and deemed to be ‘acting in a manner likely to endanger that person’s own safety or the safety of others’ for transport to hospital for medical examination (Mental Health Act, 1996: s. 28). Thus, power afforded to police via *Section 28: Police Intervention* extends that of physicians, justified by claims to enhance personal and public safety. Policing includes removing and containing people who are perceived as a threat or potential threat, incarceration, and/or application of charges. At the same time, police intervention increases the likelihood of involuntary treatment, as well as detention, incarceration, violence, and in extreme circumstances, death of people experiencing mental health and/or substance use issues (Boyd and Kerr, 2016; Wilson-Bates, 2008). Police enforcing protection through containment reinforces stigmatisation and criminalisation of people experiencing mental health issues. According to Boyd and Kerr (2016: 12), ‘concerns for public safety, amplified by the popular and overriding association of mental illness with dangerousness, have local consequences, validating increased policing of unwanted “others” in public spaces’.

The act is primarily ‘administered by health authorities, who are in turn accountable to government ministries, such as the Ministry of Health’ (Johnston, 2017: 5). Health care providers must collectively implement the act. As discussed above, the role of the physician is to decide the type and course of treatment, including the extent of measures to enforce compliance such as chemical/physical restraints and the use of seclusion.

Nurses are responsible for the implementation of the physician’s treatment plans and everyday ‘care, supervision and control’ of involuntary patients (Mental Health Act, 1996: s. 22). In hospital as well as community setting, nurses ‘actively monitor for compliance with treatment’ (Ministry of Health, 2005: 30). Contemporary practice in acute settings is largely task-oriented, with priorities oriented to ‘dispensing medication, controlling the behaviour associated with mental distress until the medication takes effect and helping the individual to adapt’ to biomedical dysfunction (Crowe and Alavi, 1999: 31; Goulter et al., 2015). Treatment compliance is ensured by coercive measures such as withholding personal clothing and access to cell phones, prolonged detainment, use of seclusion rooms, and chemical or physical restraint (Johnston, 2017; Ministry of Health, 2005). Despite the gravity of these practices, they are not addressed in the act; rather, health care providers are directed to their facility policies (Ministry of Health, 2005). In the community setting, nurses decide whether a patient should be recalled to hospital for breaching conditions of extended leave, such as when appointments are missed or medications are declined. This decision is authorised by the facility director by signing *Form 21: Director’s Warrant* (Ministry of Health, 2005: 30). Nurses also assist police in identifying the location of the patient for apprehension and transport to hospital.

Despite their significant role in protection, mention of nursing practices is almost absent in the act and the guide. Such an omission obscures their role and places it beyond scrutiny. Further nurses’ responsibilities may be in tension with their professional responsibilities to preserve autonomy, dignity, well-being, and to advocate for people for whom they provide care. Coercive psychiatric treatment does not align with contemporary and evidenced informed practices of harm reduction and trauma- and violence-informed care (Browne et al., 2015; Muskett, 2014). Rendering the nurse’s role invisible directs our gaze away from the relations of power that operate through nurses as agents of enforcement, concealing both nurses’ accountability and practice as a potential site of change.

## Operations in practice: Implementation of the *Mental Health Act*

Although the act claims to reduce harm and provide care for people experiencing mental health issues, it sustains a significant contradiction: the manner of providing protection through involuntary treatment under the act violates consent and in some situations, a patient’s perception and experience of safety. In addition, involuntary treatment under the act disproportionately affects people experiencing multiple forms of structural violence and marginalisation in its application (Czyzewski, 2011; Drescher, 2010; Kanani, 2011; Livingston, 2013; Walker, 2006).

Assumed incapacity de-legitimises concerns or objections raised by involuntary patients, undermining patient collaboration. Lack of insight justifies protectionism and deprives people of meaningful avenues for challenging certification or treatment experienced during an involuntary admission. While there are specific avenues to challenge involuntary admission and treatment, these avenues have significant barriers in relation to timeliness and accessibility (Cheung et al., 2021; Office of the Ombudsperson [British Columbia, 2019). Review panels have wait times of up to 14 days, and while patients have the right to contact private or public legal counsel, private representation is costly and public representation depends on availability. An application to court is always at the financial expense of the person. Thus, an assumption of incapacity and incompetence is a potent form of oppression that justifies ‘the removal of decision-making power’ and rejecting self-advocacy (Spagnuolo and Earle, 2017: para. 4).

While patients may take legal action against providers or institutions, they have minimal recourse. Even when subjected to permanent side-effects, maltreatment or malpractice, the right to take legal action depends on the circumstance and outcomes of involuntary treatment, and significant financial investment is needed (Groves, 2011; Johnston, 2017). Involuntary treatment under the act protects those enforcing the legislation beyond the people affected by it. A recent review by BC’s Ombudsperson furthermore found health care providers and health authorities failed to adhere to procedures outlined in the act and did not consistently complete the requisite legal forms (Office of the Ombudsperson [British Columbia, 2019]). Use of forms is now being monitored by BC’s Ministry of Health to promote oversight, accountability, and compliance with the legislation.

The discourses within the act and guide serve to maintain dominant social relationships and extend practices of governing, reinforcing a social order that dictates what is ‘normal’ and acceptable behaviour, and what is ‘abnormal’ or inappropriate behaviour. These texts both rely on and reproduce broader social values and norms of productivity, self-sufficiency, individualism, ability and rationality (Crowe, 2000). Such social values tie an individual’s value with their productivity. Ableist constructs and marginalisation sustain power relations and involuntary treatment practices formalised by the act, extending harmful psychiatric interventions into contemporary practice. These findings also offer a new perspective on alternative approaches that might reduce paternalistic and coercive practice.

## Legitimisations: Ideologies of the *Mental Health Act*

The development of the legislation from biomedical discourse attempts to construct it as neutral and objective, obscuring existing power relations within naturalisations, or presentation of pathology as a biological fact without ‘ideological effects’ (Ahmadvand, 2011: 7). Ethical application of involuntary treatment could include its use as a mechanism to support people experiencing a mental health crisis when all voluntary options have been exhausted, and only with the concurrent assessment of a person’s capacity to consent – rather than an assumption of incapacity as per the deemed consent model. Additionally, the option for secondary decision-makers and access to voluntary and preventive community mental health supports is needed.

A tautological relation operates between the two key discourses: the pathologisation of the patient underpins paternalisms in the name of protection; and protection is bolstered by pathologisation. Deciding on behalf of the patient without an obligation to obtain consent or ascertain capacity further reinforces an identity of dependence and assumes incompetence. Involuntary treatment practices are legitimised by claims to reduce the risk of harm for everyone, yet the discourse of the need for public safety entrenches ideas that people experiencing ‘mental disorders’ are both vulnerable and dangerous (McWade, 2016; Ministry of Health, 2005: 1).

Although evidence shows that mental health issues are a poor predictor of violence or crime (Canadian Mental Health Association, 2011), sensationalised popular news media have exacerbated stigma and discrimination, depicting people experiencing mental health issues as violent, unpredictable, impulsive, and criminogenic. While public interests related to safety are primarily based on misconceptions, these dominant social discourses continue to validate involuntary psychiatric treatment and police intervention in public interests of safety, including the regulation and control of ‘socially disruptive behaviour’. Entrenching these stereotypes, the act contributes to policing that disproportionately affects people experiencing mental health issues. The desire to differentiate people experiencing mental health issues from those who are not could be related to deep-seated social angst, or what Shildrick (2002) identifies as ‘the vulnerability of the self’; in witnessing another person’s struggle, we identify our own fragility.

While involuntary psychiatric treatment is meant to prevent deterioration in people experiencing mental health issues, its use to compensate for systemic shortfalls is unethical. Increased reliance on involuntary treatment is in part due to the degradation of the social safety net, and the overall lack of publicly funded community mental health services for early intervention and prevention (Health Justice, 2021). Long waitlists and restrictive eligibility criteria for public services make it difficult to access voluntary care (Mental Health Commission of Canada, 2012). Private services remain financially prohibitive for many people (Mental Health Commission of Canada, 2012) leaving few options but to wait until their mental health status deteriorates before receiving treatment. This is a costly approach for both the individual, health care system and society, leading to under-treated mental health issues, repeat admissions, and reductions in people seeking help or even avoiding mental health care due to traumatic involuntary treatment experiences (Lake and Turner, 2017).

## Conclusions and recommendations

This analysis has shown how discourses within the act and the guide extend power relations in discriminatory ways, undermining consent and increasing reliance on involuntary psychiatric treatment for people experiencing mental health issues. In so doing, this analysis exposes how the construction of involuntary patients and the need for protection operate dialectically, removing agency from people experiencing mental health issues, and normalising involuntary and coercive treatment practices (Ministry of Health, 2005: 67).

Although the guide is being revised and the forms updated, the aforementioned concerns based on the legislation will continue to structure practice in a similar manner. Discourses of equity provide an important counter to current discourses that entrench ideologies of deficit, vulnerability, dangerousness, and incapacity. An equity orientation can be applied to challenge the positioning of people experiencing mental health issues as passive, incapable patients who require the paternalistic protection imposed by the current legislation. An equity orientation addresses individual and structural issues, such as access to safe and securing housing and poverty reduction (Karban, 2016). An equity-oriented approach avoids individualistic thinking common in psychiatric discourses, while promoting nuanced understandings of mental health issues and treatment approaches that are non-coercive and anti-oppressive (Karban, 2016). Equity-oriented care can be incorporated into mental health treatment through practices grounded in trauma and violence-informed care, harm reduction, anti-racism, culturally safe care, and gender-affirming care (Browne et al., 2015).

Health policy action and amendments to legislation fall short of meaningful change in relation to structural inequity faced by people experiencing mental health issues. Health care providers are ideally positioned to call attention to and disrupt the social inequities and harmful value systems impacting people experiencing mental health issues by engaging in social justice and advocacy regarding the social determinants of health (Livingston, 2013). Advocacy for structural change is needed to influence the course of health policy processes, legislation formulation and resource allocation decisions. BC’s legislation could be reconstructed to comply with *The Charter of Rights and Freedoms (1982)* and the United Nations Convention on the Rights of Persons with Disabilities (2007), supporting mental health practice by balancing the autonomy and liberty of the individual with the need for psychiatric treatment (Gooding et al., 2018). Recommendations proposed by Community Leagal Assistant Society BC could also be implemented, including: amending the deemed consent model to promote consent rights; increased oversight and accountability of involuntary treatment processes to ensure appropriate and ethical application; periodic legal review of detentions; reimplementation of an external rights advising service to ensure people are informed of their rights; and scheduled periodic reviews of the legislation to ensure implementation of current and evidence informed approaches to involuntary psychiatric treatment (Johnston, 2017). The act could be reconstructed towards a person-centred and equity-oriented approach, requiring shared decision-making (Gooding, 2013), which could incorporate a secondary decision maker and advanced directives (Schizophrenia Society of Ontario, 2013). The role of police could also be revisited, and alternatives such as an integrated or civilian led model could be explored to reduce unnecessary force and associated harms.

Reconstruction of the texts to align with existing human rights legislation and discourses of equity has the potential to transform health care practices and structures that reproduce discourses of deficit, vulnerability and dangerousness, shifting towards promotion of the rights and safety of people experiencing mental health issues and crises. Such a reconstruction could further shift away from the unilateral power relations currently perpetuated and sustained by the legislation and ensure adequate safeguards are in place for people requiring involuntary psychiatric treatment. The construction of these texts could be written in accessible language, including translations across all languages and the development of audio, video and brail resources. This would impact the consumption of the texts by reducing the level of inferential work required for health care providers, as well as increasing transparency and awareness of involuntary practices for people who may require care. Updated texts could be made accessible online, and paper pamphlets could be distributed across health services for care providers, service-users and their families or support networks. The texts could also be distributed in schools identifying how to access preventative care, what voluntary and involuntary treatment may entail, and the rights occasioned during voluntary and involuntary care.

In order to create more accessible, equitable and prevention-oriented health systems and societies, health care providers need to work collaboratively and inter-professionally, maintaining open dialogue with service-users, families, stakeholders, politicians and policy makers. Foregrounding lived and living experience is central to providing an equitable and person-centred alternative to involuntary treatment, promoting practices that are influenced by and for the people they serve. Health care providers could advance the critical education of all who interface with the act, including analysis of the providers’ and police officers’ roles in mental health treatment, impacts of ableism in involuntary treatment and ways to mitigate coercive and unethical practices. In addition, this analysis points to the need for future research to explore the practice of health care providers, specifically how they resist or normalise involuntary treatment practices. Study of the implementation of the act could help determine the effectiveness of both voluntary and involuntary treatment approaches in BC, including extended leave. Finally, a review of global literature that analyses current least harmful approaches to involuntary psychiatric treatment offers the opportunity to generate knowledge and evidence to drive legislative and practice alternatives in BC.
